# RACK1 interaction with c-Src is essential for osteoclast function

**DOI:** 10.1038/s12276-019-0285-4

**Published:** 2019-07-29

**Authors:** Jin Hee Park, Eutteum Jeong, Jingjing Lin, Ryeojin Ko, Ji Hee Kim, Sol Yi, Youngjin Choi, In-Cheol Kang, Daekee Lee, Soo Young Lee

**Affiliations:** 1 Department of Life Science, Ewha Womans University, Seoul, 03760 Korea; 2 The Research Center for Cellular Homeostasis, Ewha Womans University, Seoul, 03760 Korea; 3 Department of Food Science & Technology, Hoseo University, Asan, 31499 Korea; 4 Department of Biological Science, College of Natural Science, BioChip Research Center, and Hoseo University, Asan, 31499 Korea

**Keywords:** Cell biology, Bone

## Abstract

The scaffolding protein receptor for activated C-kinase 1 (RACK1) mediates receptor activator of nuclear factor κΒ ligand (RANKL)-dependent activation of p38 MAPK in osteoclast precursors; however, the role of RACK1 in mature osteoclasts is unclear. The aim of our study was to identify the interaction between RACK1 and c-Src that is critical for osteoclast function. A RACK1 mutant protein (mutations of tyrosine 228 and 246 residues to phenylalanine; RACK1 Y228F/Y246F) did not interact with c-Src. The mutant retained its ability to differentiate into osteoclasts; however, the integrity of the RANKL-mediated cytoskeleton, bone resorption activity, and phosphorylation of c-Src was significantly decreased. Importantly, lysine 152 (K152) within the Src homology 2 (SH2) domain of c-Src is involved in RACK1 binding. The c-Src K152R mutant (mutation of lysine 152 into arginine) impaired the resorption of bone by osteoclasts. These findings not only clarify the role of the RACK1-c-Src axis as a key regulator of osteoclast function but will also help to develop new antiresorption therapies to prevent bone loss-related diseases.

## Bone disease: scaffolding protein essential for bone resorption

The interaction between RACK1, a so-called scaffolding protein that can assemble components of a signaling pathway into complexes, and c-Src, an enzyme involved in the re-organization of the actin cytoskeleton, is crucial for bone resorption. Previous studies have implicated RACK1 in differentiation of osteoclasts, bone cells that resorb bone, but a study led by Soo Young Lee at Ewha Womans University, Seoul, South Korea, shows that it is also required for mature osteoclast function. Mutations in RACK1 or c-Src that prevented them from binding to each other impaired the bone resorption activity of osteoclasts seeded on bone slices. Osteoclasts bearing the mutated proteins were unable to form transient actin rings, cytoskeleton structures that are typically observed during bone resorption. These findings could lead to new targeted therapies against diseases characterized by bone loss such as osteoporosis.

## Introduction

Bone is continuously remodeled by osteoblasts and osteoclasts through the balanced functions of a new bone formation and the resorption of old bone, respectively^1–3^. Osteoclasts, the only cells capable of resorbing bone, originate from the same bone marrow precursor cells within the monocyte/macrophage lineage that give rise to macrophages and dendritic cells^1–3^. Although osteoclast activity is necessary for skeletal morphogenesis and remodeling, excessive bone resorption by these cells is often associated with bone and joint diseases, such as osteoporosis and rheumatoid arthritis^3–6^.

Bone resorption by osteoclasts requires a unique cytoskeletal structure referred to as the “actin ring” or “sealing zone”^7^. Actin rings are transient structures that form only when the osteoclast is juxtaposed onto the bone. As the osteoclast detaches from the bone surface to access a new site of skeletal degradation, this ring structure disappears. Thus, the organization of the actin cytoskeleton is essential for osteoclasts to resorb bone^8^.

The non-receptor tyrosine kinase c-Src plays multiple roles in cytoskeletal regulation and cell migration^9–12^. c-Src activation is associated with the reorganization of actin within specific adhesion structures^13^. Although c-Src is ubiquitously expressed, the primary phenotype associated with the targeted disruption of *c-Src*^−/−^ mice is osteopetrosis, a condition caused by the failure to resorb bone. This phenotype results from defective osteoclasts that express high levels of c-Src^14–16^. Although mature multinucleated osteoclasts develop in *c-Src*^−/−^ mice, they are unable to form a sealing zone, an adhesive structure composed of the F-actin and integrins that is essential for bone resorption both in vivo and in vitro. These findings indicate that c-Src plays an essential role in actin dynamics and its organization in osteoclasts^13^.

The receptor for activated C-kinase 1 (RACK1) is a member of the Trp-Asp40 (WD40)-repeat protein family and exhibits a high degree of homology with the β-subunit of G proteins^17^. RACK1 was initially identified as a scaffold for protein kinase C (PKC)^18^. As a multifunctional scaffolding protein, RACK1 interacts with PKC, c-Src, and phosphodiesterase isoform PDE4D5 as well as with the cytoplasmic domain of several membrane-bound receptors, including integrin β, N-methyl-D-aspartate receptor, and insulin-like growth factor receptor I, thereby integrating the signals from various signal transduction pathways^19–25^. In a previous study, we demonstrated that RACK1-mediated activation of p38 MAPK in receptor activator of nuclear factor κΒ ligand (RANKL) signaling was necessary for osteoclast differentiation^25^; however, the role of RACK1 in osteoclast-mediated bone resorption is unclear.

In the current study, we found that the interaction between RACK1 and c-Src in osteoclasts was critical for osteoclast function. RACK1 promoted cytoskeletal reorganization in osteoclasts by functioning as a scaffold that linked c-Src to various receptors, including RANK and αVβ3 integrin. Our findings provide insights into the mechanism by which RANK mediates cytoskeletal reorganization during the process of bone resorption.

## Materials and methods

### Mice and cells

Bone marrow-derived macrophages (BMMs) derived from 6–8-week-old male C57BL/6 mice (The Jackson Laboratory) were prepared as previously described^26^. The 293T cell line was used for the protein–protein interaction experiments. All animal experiments were approved by the Institutional Animal Care and Use Committee of Ewha Laboratory Animal Genomics Center and were conducted in accordance with the approved guidelines.

### Plasmids

The pcDNA3.1 vector encoding HA-RACK1 was provided by M.J.W. (University of Virginia Health System, Charlottesville, VA, USA). The empty pMX-puro vector, pMX-puro-WT-RACK1, pMX-puro-control shRNA, and pMX-puro-shRACK1 were described previously^25^. Mutant constructs (RACK1 Y228F/Y246F and c-Src K152R) were generated using site-directed mutagenesis with QuikChange reagents (Stratagene, La Jolla, CA, USA). Recombinant retroviral vectors encoding RACK1 Y228F/Y246F, c-Src WT, and c-Src K152R were generated by subcloning the corresponding cDNAs into the retroviral pMX-puro vector.

### Reagents

Recombinant human M-CSF was purchased from R&D Systems (Minneapolis, MN, USA). RANKL was obtained from Peprotech EC (London, England). The antibody against RACK1 used for western blotting was purchased from BD Biosciences (San Jose, CA, USA). Anti-c-Src was purchased from Abcam Biotechnology (Cambridge, UK). Anti-phospho-c-Src and anti-HA were purchased from Cell Signaling Technology (Beverly, MA, USA). The anti-RACK1 antibody used for immunoprecipitation, as well as anti-NFATc1, anti-4G10 and anti-β-actin, was obtained from Santa Cruz Biotechnology, Inc. (Dallas, TX, USA). Anti-Atp6v0d2 was provided by Y.C. (University of Pennsylvania, Philadelphia, PA, USA).

### Transfection experiments and protein analysis

Cells were transfected with expression vectors using PEI transfection reagent (Sigma-Aldrich). For the coexpression assays, 293T cells were transfected with the indicated expression vectors. The transfected cells were analyzed using western blotting. The cell lysates were immunoprecipitated with the indicated antibodies and subsequently analyzed using western blotting.

### Retrovirus preparation

Retroviruses were prepared by transfecting PLAT-E packaging cells with empty pMX-puro vector, pMX-puro-WT-RACK1, pMX-puro-MT-RACK1, pMX-puro-control shRNA, or pMX-puro-shRACK1 using the PEI transfection reagent (Sigma-Aldrich). BMMs were infected with the retroviruses as previously described^25^. The pMX-puro vector and PLAT-E cells were kindly provided by T.K. (University of Tokyo, Tokyo, Japan). After infection, the BMMs were cultured overnight, detached with trypsin/ethylenediaminetetraacetic acid, and further cultured in the presence of 30 ng/mL M-CSF and 2 μg/mL puromycin for 2 days. Puromycin-resistant BMMs were induced to differentiate by culturing the cells with 30 ng/mL M-CSF and 100 ng/mL RANKL for an additional 3–4 days.

### In vitro osteoclast differentiation

The cells were fixed and stained for the presence of tartrate-resistant acid phosphatase (TRAP) using a TRAP staining Kit (Sigma-Aldrich). Osteoclast-like cells were defined as pink TRAP-positive multinucleated cells (i.e., more than three nuclei). The results of the osteoclast formation assays represent the mean of three independent experiments performed in triplicate ±standard deviation (SD) of the mean.

### Actin ring reformation

Actin ring staining and quantitation were conducted as previously described^27^. Briefly, mature osteoclasts were seeded on bone slices and cultured with 30 ng/mL M-CSF and 100 ng/mL RANKL for 2 days to induce the osteoclast phenotype. The actin rings were disrupted by washing the bone slices twice with cold cytokine-free medium, after which the slices were incubated in osteoclast differentiation medium for 120 min. The slices were fixed and stained with Alexa Fluor 488-phalloidin. The osteoclasts were identified using a Zeiss Axioplan II fluorescence microscope (Zeiss). Osteoclasts were defined as cells containing at least three nuclei. The number of osteoclasts on each coverslip was noted, and a blinded investigator scored each osteoclast according to its type of actin cytoskeletal structure.

### Bone resorption assay

Mature osteoclasts were seeded on bone slices and cultured with 30 ng/mL M-CSF and 100 ng/mL RANKL for 3 days. The bone slices were mechanically agitated to remove the cells and then stained with hematoxylin solution and Gill no. 3 for 10 min. Quantitative analysis of the′ resorbed pit area was conducted using ImageJ (NIH, Bethesda, MD, USA). Four bone slices were measured under each experimental condition.

### Real-time quantitative polymerase chain reaction

BMMs were cultured with M-CSF in the presence or absence of RANKL for the indicated period of time. Total RNA was extracted using TRIzol (Invitrogen, Paisley, Scotland, UK) according to the manufacturer’s instructions. Total RNA was reverse transcribed into cDNA using an M-MLV Kit (SolGent, Seoul, Korea). Polymerase chain reaction (PCR) amplification was conducted using a SYBR Green Master Kit (Kapa Biosystems, Woburn, MA, USA). The ABI PRISM 7300 system (Applied Biosystems, Foster City, CA, USA) was used to amplify DNA and detect the resulting products. Each experiment was conducted in triplicate, and the expression levels of the target genes were normalized to those of actin. The melting curve was analyzed to ensure that only the desired PCR product was present. The gene-specific primers for real-time PCR were as follows: RACK1 sense, 5ʹ-GCCTCTGGGATCTCACAAC-3ʹ and antisense, 5ʹ-AACTTTATGGTCTTGTCTCGGG-3ʹ; Src sense, 5ʹ-ACCACCTTTGTGGCC CTCTATG-3ʹ and antisense, 5ʹ-GCCACCAGTCTCCCTCTGTGTT-3ʹ; NFATc1 sense, 5ʹ-CCAGAAAATAACATGCGAGCC-3ʹ and antisense, 5ʹ-GTGGGATGTGAACTCGGAAG-3ʹ; Actin sense, 5ʹ-AGATGTGGATCAGCAAGCAG-3ʹ and antisense, 5ʹ-GCGCAAGTTAGGTTTTGTCA-3ʹ. Data were normalized to β-actin mRNA expression.

### Western blot analysis

**Fig. 1:**
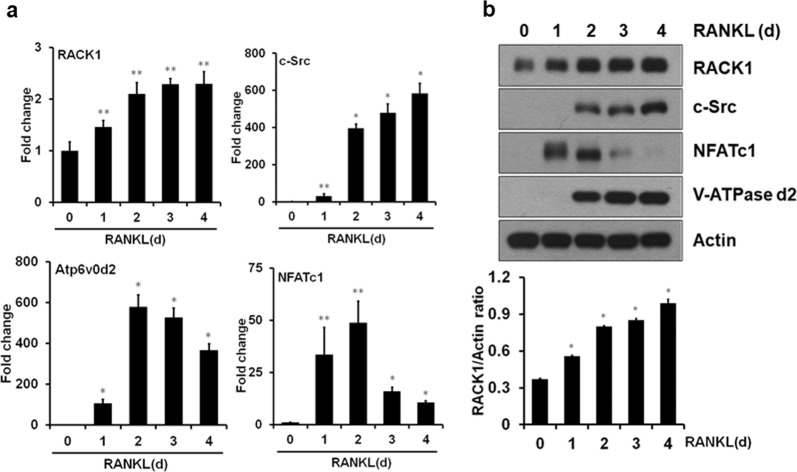
RACK1 is upregulated during RANKL-induced osteoclastogenesis. Bone marrow-derived macrophages (BMMs) were cultured with 30 ng/mL M-CSF and 100 ng/mL RANKL for the indicated period of time. **a** RACK1, c-Src, NFATc1, and V-ATPase d2 mRNA levels were analyzed using real-time PCR. Data are presented as the mean ± SD of three independent experiments. *P < 0.01, **P < 0.05. **b** RACK1, c-Src, NFATc1, and V-ATPase d2 protein levels in whole cell lysates were analyzed by western blotting with antibodies specific for the indicated proteins. The ratio of RACK1 to actin was quantified from three independent experiments. *P < 0.01, **P < 0.05

**Fig. 2:**
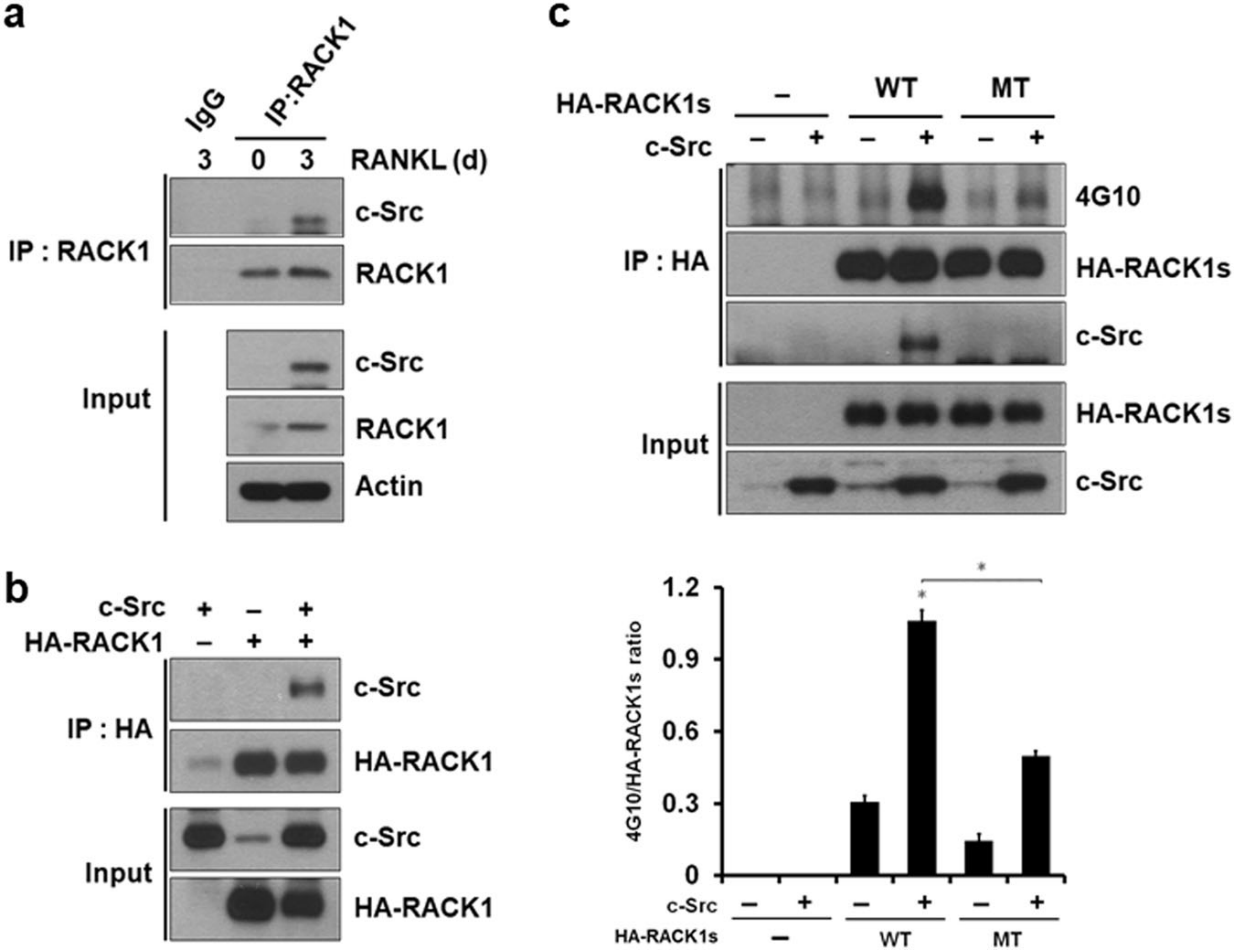
RACK1 associates with c-Src in osteoclasts. **a** BMMs were incubated in the presence or absence of 100 ng/mL RANKL for 3 days. The cells were lysed, and endogenous RACK1 was immunoprecipitated using anti-RACK1. The immunoprecipitates were analyzed using western blotting with anti-RACK1 and anti-c-Src. **b** First, 293T cells were transfected with plasmids expressing HA-RACK1 or c-Src as indicated. RACK1 was immunoprecipitated using anti-HA, and the immunoprecipitates and cell lysates were analyzed using western blotting with the indicated antibodies. The levels of exogenously expressed HA-RACK1 and c-Src in the cell lysates (Input) were assessed using western blotting. **c** The 293T cells were transfected with the indicated expression vectors. RACK1 was immunoprecipitated from cell lysates using anti-HA. The precipitated complexes and cell lysates were analyzed using western blotting with antibodies specific for the indicated proteins. The ratio of 4G10 to HA-RACK1 was quantified in each of three independent experiments. *P < 0.01, **P < 0.05. Western blots in **a**–**c** are representative of three independent experiments

**Fig. 3:**
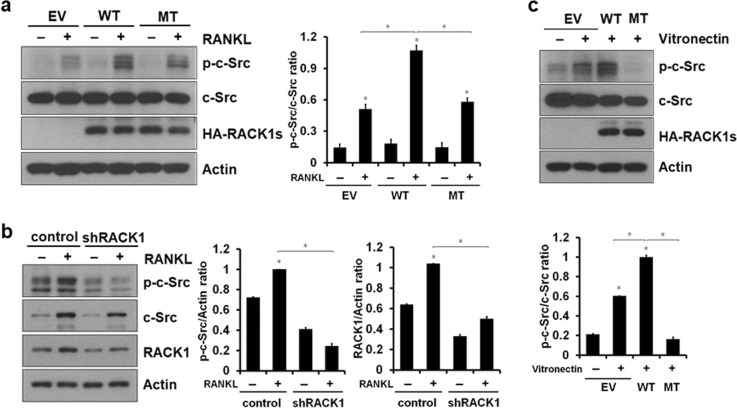
RACK1 regulates RANKL-induced c-Src activation. **a** Mature osteoclasts generated from BMMs were serum-starved and stimulated with 200 ng/mL RANKL for 20 min. Whole cell lysates were analyzed using western blotting with anti-phospho-c-Src, anti-c-Src, anti-HA, and anti-actin. The ratio of p-c-Src to total c-Src proteins was quantified from each of three independent experiments. *P < 0.01. **b** BMMs transduced with pMX-puro control shRNA (control) or pMX-puro-shRACK1 (shRACK1) retrovirus were cultured for 3 days with 30 ng/mL M-CSF and 100 ng/mL RANKL to generate mature osteoclasts. Protein levels were analyzed using western blotting with antibodies specific for the indicated proteins. The ratios of p-c-Src to actin and RACK1 to actin were quantified from each of three independent experiments. *P < 0.01. **c** Mature osteoclasts generated from BMMs were cultured with 30 ng/mL M-CSF and 100 ng/mL RANKL for 4 days. After the cells were removed, they were either maintained in suspension or plated on a vitronectin-coated dish for 30 min. Cell lysates were analyzed using western blotting with antibodies against anti-phospho-c-Src, anti-c-Src, anti-HA, and anti-actin. The ratio of p-c-Src to total c-Src proteins was quantified in each of three independent experiments. *P < 0.01. Western blots in **a**–**c** are representative of three independent experiments

**Fig. 4:**
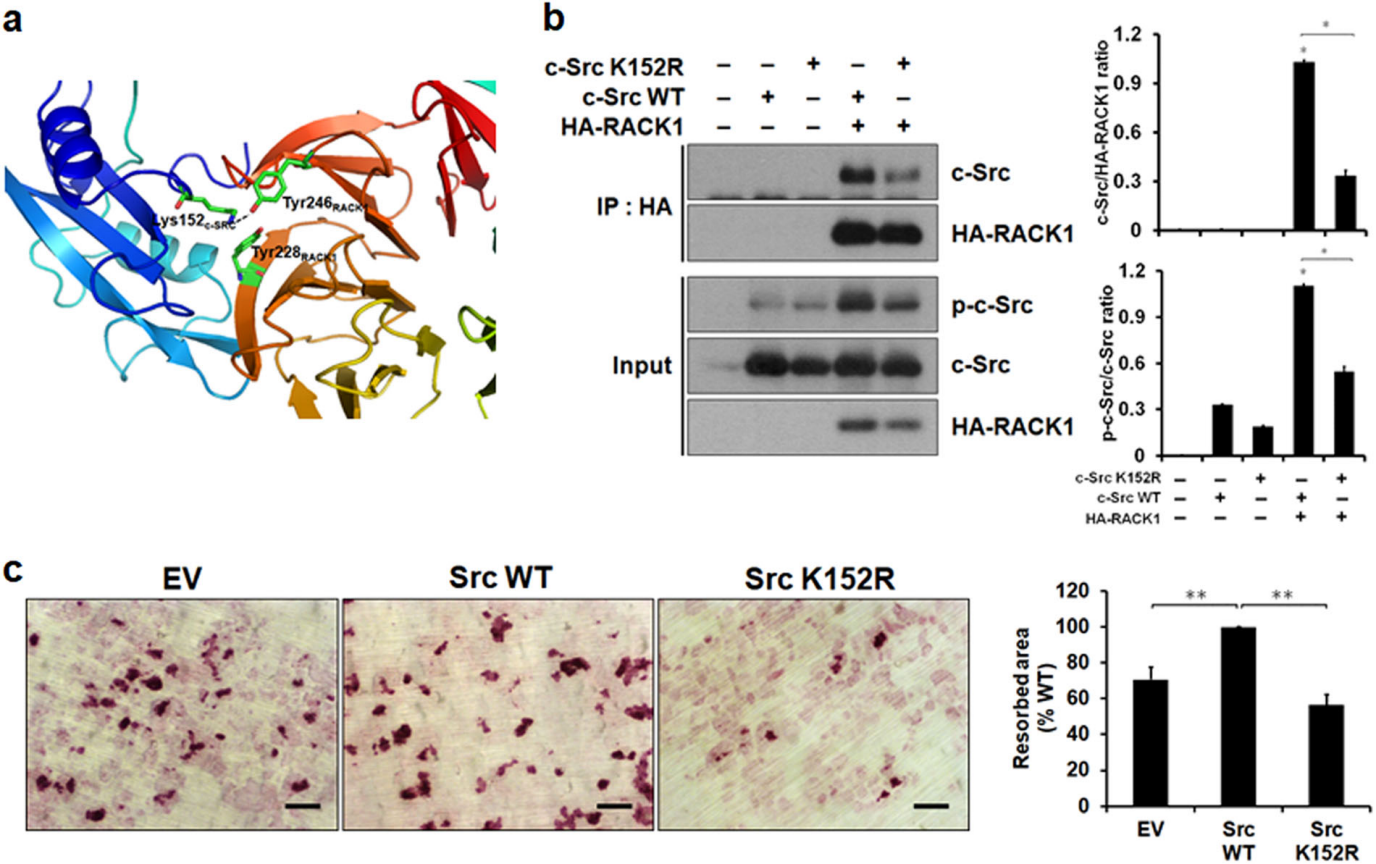
c-Src K152R mutation affects osteoclastic bone resorption. **a** Model of the interaction between the SH2 domain of c-Src and RACK1. Key interacting residues were rendered as a space-filling structure. **b** First, 293T cells were transfected with the indicated expression vectors. RACK1 was immunoprecipitated from cell lysates using anti-HA. The precipitated complexes and cell lysates were analyzed using western blotting. The ratios of c-Src to HA-RACK1 and p-c-Src to c-Src were quantified in each of three independent experiments. *P < 0.01. **c** BMMs transduced with pMX-puro empty vector (EV), pMX-puro-WT-c-Src (Src WT), or pMX-puro-K152R-c-Src (Src K152R) were cultured for 3 days with 30 ng/mL M-CSF and 100 ng/mL RANKL to generate mature osteoclasts. Mature osteoclasts were seeded on bone slices and cultured for 3 days. The cells were then removed, and the bone slices were stained. Scale bar, 50 μm. Right: images of the stained sections were used to calculate the resorption pit areas. Data are presented as the mean ± SD of three independent experiments. *P < 0.01, **P < 0.05

The cells were lysed in a buffer containing 20 mM HEPES (pH 7.0), 150 mM NaCl, 1% Triton X-100, 10% glycerol, proteinase inhibitors (1 mM PMSF and 1 μg/mL leupeptin and aprotinin) and phosphatase inhibitors (1 mM NaVO4 and 1 mM NaF) after vortexing on ice for 30 min. After centrifuging for 20 min, the supernatants were boiled in 6X SDS sample buffer containing 0.6 M DTT. Cell lysates or immunoprecipitated proteins were separated using 10% SDS-polyacrylamide gels and electrotransferred onto a PVDF membrane (Millipore, Billerica, MA, USA). The membranes were blocked with 5% bovine serum albumin in Tris-buffered saline containing 0.1% Tween-20 and were immunoblotted with primary antibodies against RACK1, c-Src, phospho-c-Src, 4G10 (1:1000), HA (1:2000), NFATc1 (1:500), β-actin (1:5000), and Atp6v0d2 (1:10000) and secondary antibodies conjugated to HRP (1:5000). Proteins were detected using an ECL detection Kit (Bio-Rad Laboratories, Hercules, CA, USA). Representative western blots and quantification (shown in the bar graph) of the indicated protein/control ratio in the cell lysates using ImageJ are shown in Figs. 1b; 2c; 3a–c and 4b.

### Protein–protein docking

Three-dimensional structures of RACK1 (PDB id 4AOW) and the SH2 domain of c-Src (PDB ID 1FBZ) were obtained from the Protein Data Bank. Each protein structure was docked using the ZDOCK server^28^ and the web-based protein–protein docking simulator^28^. During this modeling process, ZDOCK 3.0.2 was used for the protein complex. The top-scoring pose was selected from the predicted structures.

### Statistical analysis

Data are expressed as the mean ± SD of at least three independent experiments. Statistical analyses were performed using Student’s *t*-test to analyze differences among the groups. *P < 0.01 and **P < 0.05 were considered statistically significant.

## Results

### Expression of RACK1 during RANKL-induced osteoclastogenesis

RACK1 is highly expressed in all mammalian cells at relatively constant levels^24,29^; however, RANKL stimulation during osteoclast formation promoted a gradual increase in RACK1 expression at both the mRNA and protein levels (Fig. 1a, b). Consistent with previous reports^6,30^, we found that NFATc1 was upregulated at both the mRNA and protein levels 1 day after RANKL stimulation. NFATc1 mRNA and protein levels were at their maximum 2 days after RANKL stimulation and then declined (Fig. 1a, b). The upregulation of NFATc1 expression was accompanied by the upregulation of c-Src and Atp6V0d2, two known downstream targets of NFATc1^31,32^. NFATc1, c-Src, and Atp6V0d2 protein levels were undetectable in BMMs, the cells that give rise to osteoclasts, but their levels increased during osteoclast differentiation. The expression pattern of RACK1 during RANKL-induced osteoclast formation suggests that RACK1 plays a role in the signaling pathway that mediates osteoclast function.

### RACK1 interacts with c-Src in osteoclasts

Previous studies have shown that the interaction between RACK1 and c-Src regulates the proliferation of cancer cells^33^. To investigate the molecular link between RACK1 and c-Src in osteoclasts, we first examined whether these two proteins associate in this context. The results of an immunoprecipitation assay using an antibody against RACK1 demonstrated that endogenous RACK1 interacts with c-Src in osteoclasts (Fig. 2a), but because BMMs do not express c-Src, the two proteins do not coimmunoprecipitate. This interaction was further confirmed by the observation that ectopically expressed c-Src coimmunoprecipitates with RACK1 in 293T cells (Fig. 2b). Consistent with a previous report^34^, c-Src did not phosphorylate a RACK1 mutant protein in which both tyrosine residues at positions 228 and 246 were replaced with phenylalanine (Y228F/Y246F) (Fig. 2c). Furthermore, the RACK1 mutant protein did not bind to c-Src, which suggests that the interaction between c-Src and RACK1 is mediated by tyrosine phosphorylation on Y228 and/or Y246 (Fig. 2c).

### Y228F/Y246F mutations in RACK1 do not influence RANKL-induced osteoclastogenesis

We previously demonstrated that RACK1 functions as a scaffolding protein in the p38 MAP kinase pathway, indicating the link between the RANKL signaling cascade and osteoclastogenesis^25^. To investigate the effect of the RACK1 mutation (Y228F/Y246F) on RANKL-induced osteoclast formation, we overexpressed wild-type (WT) or mutant RACK1 in BMMs. The overexpression of either the WT or mutant RACK1 enhanced the formation of large multinucleated osteoclasts (Supplementary Fig. S1a, b). Moreover, NFATc1 levels in the cells that overexpressed mutant RACK1 were similar to those in cells expressing WT RACK1 (Supplementary Fig. S1c). These results suggest that the interaction between c-Src and RACK1 and the c-Src-mediated phosphorylation of RACK1 are not involved in osteoclast differentiation.

### RACK1 regulates actin ring and pit formation through interaction with c-Src

**Fig. 5:**
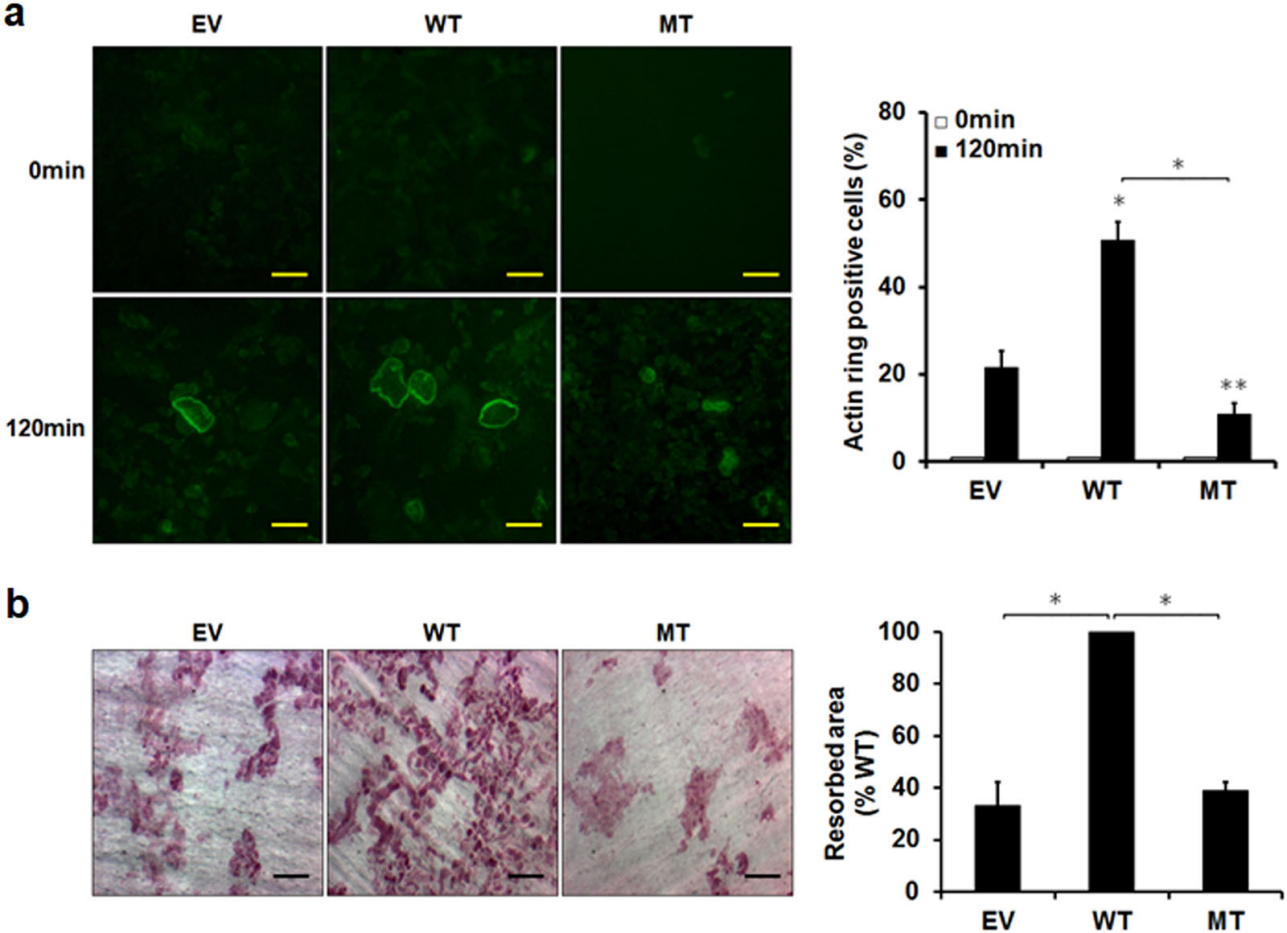
RACK1 regulates osteoclast cytoskeleton organization and bone resorption through its interaction with c-Src. BMMs transduced with pMX-puro empty vector (EV), pMX-puro-WT-RACK1 (WT), or pMX-puro-MT-RACK1 (MT) were cultured for 3 days with 30 ng/mL M-CSF and 100 ng/mL RANKL to generate mature osteoclasts. **a** Mature osteoclasts were seeded on bone slices and cultured for 2 days. Actin rings were disrupted by washing the cells with phosphate-buffered saline and then treating them with 100 ng/mL RANKL for 120 min. To visualize the actin rings, the bone slices were fixed and stained with Alexa Fluor 488-phalloidin. Scale bar, 50 μm. Right: four fields were randomly selected, and the number of intact actin rings was counted (~50–100) by two independent assessors. Three bone slices were measured under each experimental condition. The percentage of osteoclasts with actin rings is indicated as actin ring positive cells (%). **b** Mature osteoclasts were seeded on bone slices and cultured for 3 days. The cells were then removed, and the bone slices were stained. Scale bar, 50 μm. Right: images of the stained sections were used to calculate the area of resorption pits. Data are presented as the mean ± SD of three independent experiments. *P < 0.01, **P < 0.05

Based on the observation that RACK1 and c-Src within osteoclasts interact, we hypothesized that RACK1 might regulate c-Src activity in these cells. Because c-Src plays an essential role in cytoskeletal organization by osteoclasts^9,35^, we examined the effect of RACK1 overexpression on the formation of the actin ring, a cytoskeletal structure essential for optimal osteoclast-mediated bone resorption^36,37^. To this end, we generated mature osteoclasts on dentin discs. As shown in Fig. 5a, the number of actin rings significantly increased in the RANKL-stimulated cells that overexpressed WT RACK1 compared with the control cells; however, RANKL-stimulated cells that overexpressed mutant RACK1 failed to promote actin ring formation. Consistent with these results, the bone resorption activity of the RACK1-overexpressing osteoclasts significantly increased compared with that in the control osteoclasts (Fig. 5b), whereas the bone resorption activity of osteoclasts overexpressing mutant RACK1 was markedly decreased compared with that in the control osteoclasts. These results suggest that the interaction between RACK1 and c-Src in osteoclasts is necessary for osteoclast-mediated actin ring formation and bone resorption.

### RACK1 mediates RANKL- and integrin-mediated c-Src phosphorylation

To further elucidate the function of the interaction between RACK1 and c-Src in osteoclasts, we examined the effect of RACK1 on c-Src phosphorylation in cells stimulated with RANKL and integrin. The overexpression of WT RACK1, but not the overexpression of mutant RACK1, enhanced RANKL-induced c-Src phosphorylation (Fig. 3a). A similar result was observed in RACK1-knockdown osteoclasts (Fig. 3b). Because αVβ3 integrin-induced c-Src phosphorylation is a key step in actin ring formation^38^, we investigated the effect of RACK1 on integrin-mediated c-Src phosphorylation. To this end, we plated osteoclasts on vitronectin-coated plates for 15 min to promote integrin clustering. c-Src phosphorylation levels significantly increased in cells overexpressing WT RACK1 compared with control cells, whereas c-Src phosphorylation levels strongly decreased in cells overexpressing mutant RACK1 (Fig. 3c). Interestingly, neither WT nor mutant RACK1 affected M-CSF-induced c-Src phosphorylation (Supplementary Fig. S2a). Together, these results suggest that RACK1 promotes RANKL- and integrin-induced c-Src phosphorylation in osteoclasts.

### The K152 residue of c-Src is involved in the RACK1 interaction

A computational protein–protein docking study predicted that RACK1 was bound to the SH2 domain of c-Src through specific hydrogen bonding between Y246 in RACK1 and the K152 residue in c-Src. Furthermore, K152 showed favorable van der Waals interactions with Y228 in RACK1 (Fig. 4a). To confirm that the K152 residue in c-Src is responsible for RACK1 binding, we mutated K152 into arginine (K152R) and tested its interaction with RACK1. The c-Src K152R mutant association with RACK1 was impaired, while that of WT c-Src was not (Fig. 4b). Importantly, the bone resorption activity of the c-Src K152R mutant in the osteoclasts significantly decreased compared with that in the WT c-Src (Fig. 4c). These observations suggest a model in which RACK1 interacts with K152 within the SH2 domain of c-Src. Furthermore, this interaction is necessary for the bone resorption activity of osteoclasts.

## Discussion

Although the involvement of c-Src in the regulation of RANK signaling has been documented^14,35,38^, the precise regulatory mechanism of c-Src in this context has remained elusive. Previous studies have suggested that the regulation of c-Src in RANK relies on TRAF6^39,40^, a signaling adaptor common to the IL-1R/TLR family and TNFR superfamily;^27,41,42^ however, pathways independent of TRAF6 have also been implicated in this process^43,44^. The present study demonstrated that RACK1 plays a key role in RANK-mediated c-Src activation and that the phosphorylation of RACK1 by c-Src enhances RANKL-induced actin ring formation in osteoclasts. These findings represent a potential mechanism of the underlying activation of the RANK signaling cascade by the RACK1-c-Src axis. Our findings also provide insight into the mechanism underlying the crosstalk between c-Src and RACK1 in response to RANKL stimulation.

In osteoclasts, c-Src is essential in the regulation of membrane ruffling and the formation of actin rings that facilitate adhesion to the bony matrix and bone resorption^45,46^. Similarly, multiple factors, including M-CSF, integrin, and RANKL, rapidly induce changes in cytoskeletal organization to promote cell spreading, cell motility^47^, and actin ring formation^48^. c-Src is a key component of the signaling pathways that regulate the osteoclast cytoskeleton in response to M-CSF, integrin, and RANKL^49,50^, which suggests that these factors most likely regulate the osteoclast cytoskeleton through c-Src; however, the mechanism underlying c-Src regulation in response to specific stimuli in the osteoclasts remains unclear. We propose that the scaffolding protein RACK1 is a key component of the c-Src pathway in osteoclasts and that RACK1 links c-Src signaling to RANKL and integrin but not to M-CSF.

The recruitment and activation of c-Src into the RANK receptor most likely involves a multistep process. First, RANKL stimulation induces RANK oligomerization to recruit TRAF6^40^. RACK1 is subsequently recruited to the receptor complex by TRAF6^25^. RACK1 either directly recruits c-Src to the receptor complex, and/or TRAF6 activates c-Src and induces it to interact with other signaling molecules. In this context, RACK1 most likely functions as an important regulator that selectively recruits signaling modules to c-Src in response to specific stimuli. The scaffolding function of RACK1 is reminiscent of the role of β-arrestin 1 in recruiting c-Src to the β2 adrenergic receptor, a G protein-coupled receptor^51^. Receptors that lack intrinsic tyrosine kinase activity might need adaptor or scaffolding proteins, such as TRAF6 and RACK1, to recruit and activate Src family kinases.

The expression pattern of RACK1 during osteoclast formation and osteoclast-mediated bone resorption supports the hypothesis that RACK1 participates in the signaling pathways that mediate these processes. Multiple studies have demonstrated that the association between RACK1 and β1/β2 integrin and between RACK1 and c-Src regulates cell adhesion and cell motility in cancer cells^49–51^. Notably, because osteoclast adhesion and spreading play important roles in bone resorption^7,52^, the role of RACK1 in these processes merits further investigation.

In conclusion, we propose that RACK1 functions as a scaffolding protein in the c-Src pathway, thereby linking it to the RANKL-signaling cascade. c-Src phosphorylates RACK1 on Tyr 228 and/or Tyr 246. Tyr 228 and Tyr 246 are highly conserved residues located in the sixth tryptophan-aspartic acid repeat, which in turn interacts with the SH2 domain of c-Src. We speculate that RACK1 is an important c-Src substrate that transduces signals downstream of RANK and is involved in the regulation of c-Src activation and osteoclast cytoskeletal reorganization.

## Supplementary information

Supplementary Information

## References

[1] Kim N, Takami M, Rho J, Josien R, Choi Y (2002). A novel member of the leukocyte receptor complex regulates osteoclast differentiation. J. Exp. Med..

[2] Ikeda K, Takeshita S (2014). Factors and mechanisms involved in the coupling from bone resorption to formation: how osteoclasts talk to osteoblasts. J. Bone Metab..

[3] Goldring SR, Gravallese EM (2000). Mechanisms of bone loss in inflammatory arthritis: diagnosis and therapeutic implications. Arthritis Res..

[4] Martin TJ (2014). Bone biology and anabolic therapies for bone: current status and future prospects. J. Bone Metab..

[5] Takayanagi H (2000). T-cell-mediated regulation of osteoclastogenesis by signalling cross-talk between RANKL and IFN-gamma. Nature.

[6] Park SJ (2016). Sirt6 cooperates with Blimp1 to positively regulate osteoclast differentiation. Sci. Rep..

[7] Teitelbaum SL (2000). Bone resorption by osteoclasts. Science.

[8] Teitelbaum SL (2011). The osteoclast and its unique cytoskeleton. Ann. N. Y. Acad. Sci..

[9] Sanjay A (2001). Cbl associates with Pyk2 and Src to regulate Src kinase activity, alpha(v) beta(3) integrin-mediated signaling, cell adhesion, and osteoclast motility. J. Cell Biol..

[10] Frame MC (2004). Newest findings on the oldest oncogene; how activated src does it. J. Cell Sci..

[11] Playford MP, Schaller MD (2004). The interplay between Src and integrins in normal and tumor biology. Oncogene.

[12] Alper O, Bowden ET (2005). Novel insights into c-Src. Curr. Pharm. Des..

[13] Destaing O (2008). The tyrosine kinase activity of c-Src regulates actin dynamics and organization of podosomes in osteoclasts. Mol. Biol. Cell.

[14] Soriano P, Montgomery C, Geske R, Bradley A (1991). Targeted disruption of the c-src proto-oncogene leads to osteopetrosis in mice. Cell.

[15] Horne WC (1992). Osteoclasts express high levels of pp60c-src in association with intracellular membranes. J. Cell Biol..

[16] Lowell CA, Soriano P (1996). Knockouts of Src-family kinases: stiff bones, wimpy T cells, and bad memories. Genes Dev..

[17] McCahill A, Warwicker J, Bolger GB, Houslay MD, Yarwood SJ (2002). The RACK1 scaffold protein: a dynamic cog in cell response mechanisms. Mol. Pharm..

[18] Ron D (1994). Cloning of an intracellular receptor for protein kinase C: a homolog of the beta subunit of G proteins. Proc. Natl Acad. Sci. USA.

[19] Mamidipudi V, Cartwright CA (2009). A novel pro-apoptotic function of RACK1: suppression of Src activity in the intrinsic and Akt pathways. Oncogene.

[20] Bird RJ, Baillie GS, Yarwood SJ (2010). Interaction with receptor for activated C-kinase 1 (RACK1) sensitizes the phosphodiesterase PDE4D5 towards hydrolysis of cAMP and activation by protein kinase C. Biochem J..

[21] Besson A, Wilson TL, Yong VW (2002). The anchoring protein RACK1 links protein kinase Cepsilon to integrin beta chains. Requirements for adhesion and motility. J. Biol. Chem..

[22] Wang J (2010). Long-lasting adaptations of the NR2B-containing NMDA receptors in the dorsomedial striatum play a crucial role in alcohol consumption and relapse. J. Neurosci..

[23] Kiely PA, Sant A, O’Connor R (2002). RACK1 is an insulin-like growth factor 1 (IGF-1) receptor-interacting protein that can regulate IGF-1-mediated Akt activation and protection from cell death. J. Biol. Chem..

[24] Adams DR, Ron D, Kiely PA (2011). RACK1, amultifaceted scaffolding protein: Structure and function. Cell Commun. Signal..

[25] Lin, J., Lee, D., Choi, Y. & Lee, S. Y. The scaffold protein RACK1 mediates the RANKL-dependent activation of p38 MAPK in osteoclast precursors. *Sci. Signal.* **8**, ra54 (2015). 10.1126/scisignal.2005867 PMC4492518 26038599

[26] Jang HD (2011). Inactivation of glycogen synthase kinase-3beta is required for osteoclast differentiation. J. Biol. Chem..

[27] Ko R, Park JH, Ha H, Choi Y, Lee SY (2015). Glycogen synthase kinase 3beta ubiquitination by TRAF6 regulates TLR3-mediated pro-inflammatory cytokine production. Nat. Commun..

[28] Pierce BG (2014). ZDOCK server: interactive docking prediction of protein-protein complexes and symmetric multimers. Bioinformatics.

[29] Robles MS, Boyault C, Knutti D, Padmanabhan K, Weitz CJ (2010). Identification of RACK1 and protein kinase Calpha as integral components of the mammalian circadian clock. Science.

[30] Choi HK (2013). Early estrogen-induced gene 1, a novel RANK signaling component, is essential for osteoclastogenesis. Cell Res..

[31] Kim K, Lee SH, Ha Kim J, Choi Y, Kim N (2008). NFATc1 induces osteoclast fusion via up-regulation of Atp6v0d2 and the dendritic cell-specific transmembrane protein (DC-STAMP). Mol. Endocrinol..

[32] Song I (2009). Regulatory mechanism of NFATc1 in RANKL-induced osteoclast activation. FEBS Lett..

[33] Mamidipudi V (2007). RACK1 inhibits colonic cell growth by regulating Src activity at cell cycle checkpoints. Oncogene.

[34] Chang BY, Harte RA, Cartwright CA (2002). RACK1: a novel substrate for the Src protein-tyrosine kinase. Oncogene.

[35] Miyazaki T (2004). Src kinase activity is essential for osteoclast function. J. Biol. Chem..

[36] Lakkakorpi PT, Vaananen HK (1991). Kinetics of the osteoclast cytoskeleton during the resorption cycle in vitro. J. Bone Min. Res..

[37] Lakkakorpi PT, Vaananen HK (1996). Cytoskeletal changes in osteoclasts during the resorption cycle. Microsc. Res. Tech..

[38] Zou W (2007). Syk, c-Src, the alphavbeta3 integrin, and ITAM immunoreceptors, in concert, regulate osteoclastic bone resorption. J. Cell Biol..

[39] Wong BR (1999). TRANCE, a TNF family member, activates Akt/PKB through a signaling complex involving TRAF6 and c-Src. Mol. Cell.

[40] Armstrong AP (2002). A RANK/TRAF6-dependent signal transduction pathway is essential for osteoclast cytoskeletal organization and resorptive function. J. Biol. Chem..

[41] Kim JM (2005). Induction of proinflammatory mediators requires activation of the TRAF, NIK, IKK and NF-kappaB signal transduction pathway in astrocytes infected with Escherichia coli. Clin. Exp. Immunol..

[42] Kobayashi T, Walsh MC, Choi Y (2004). The role of TRAF6 in signal transduction and the immune response. Microbes Infect..

[43] Kim H (2009). Selective inhibition of RANK blocks osteoclast maturation and function and prevents bone loss in mice. J. Clin. Investig..

[44] Izawa T (2012). c-Src links a RANK/alphavbeta3 integrin complex to the osteoclast cytoskeleton. Mol. Cell Biol..

[45] Boyce BF, Yoneda T, Lowe C, Soriano P, Mundy GR (1992). Requirement of pp60c-src expression for osteoclasts to form ruffled borders and resorb bone in mice. J. Clin. Investig..

[46] Schwartzberg PL (1997). Rescue of osteoclast function by transgenic expression of kinase-deficient Src in src-/- mutant mice. Genes Dev..

[47] Fuller K, Wong B, Fox S, Choi Y, Chambers TJ (1998). TRANCE is necessary and sufficient for osteoblast-mediated activation of bone resorption in osteoclasts. J. Exp. Med..

[48] Burgess TL (1999). The ligand for osteoprotegerin (OPGL) directly activates mature osteoclasts. J. Cell Biol..

[49] Sato S (2005). Essential function for the kinase TAK1 in innate and adaptive immune responses. Nat. Immunol..

[50] Shim JH (2005). TAK1, but not TAB1 or TAB2, plays an essential role in multiple signaling pathways in vivo. Genes Dev..

[51] Luttrell LM (1999). Beta-arrestin-dependent formation of beta2 adrenergic receptor-Src protein kinase complexes. Science.

[52] Boyle WJ, Simonet WS, Lacey DL (2003). Osteoclast differentiation and activation. Nature.

