# Supplementary material for: RACK1 interaction with c-Src is essential for osteoclast function
Source: Exp Mol Med. 2019 Jul 29;51(7):86. doi: 10.1038/s12276-019-0285-4 (PMC6802652; doi:10.1038/s12276-019-0285-4)
Supplement: Supplementary file 1 — Supplementary Information [file 12276_2019_285_MOESM1_ESM.docx]

**Supplementary Information**

**RACK1 interaction with c-Src is essential for osteoclast function**

Jin Hee Park^1,2,*^, Eutteum Jeong^1,2,*^, Jingjing Lin^1,2,*^, Ryeojin Ko^1,2^, Ji Hee Kim^1^, Sol Yi^1^, Young Jin Choi^3^, In-Cheol Kang^3^, Daekee Lee^1^, and Soo Young Lee^1,2,§^

^1^Department of Life Science, Ewha Womans University, Seoul 03760, Korea.

^2^The Research Center for Cellular Homeostasis, Ewha Womans University, Seoul 03760, Korea

^3^Department of Food Science & Technology, Hoseo University, Asan 31499, Korea.

^4^Department of Biological Science, College of Natural Science, BioChip Research Center, and Hoseo University, Asan 31499, Korea.

^§^Correspondence should be addressed to S.Y.L.

Tel:82-2-3277-3770; Fax:82-2-3277-3760; E-mail: leesy@ewha.ac.kr


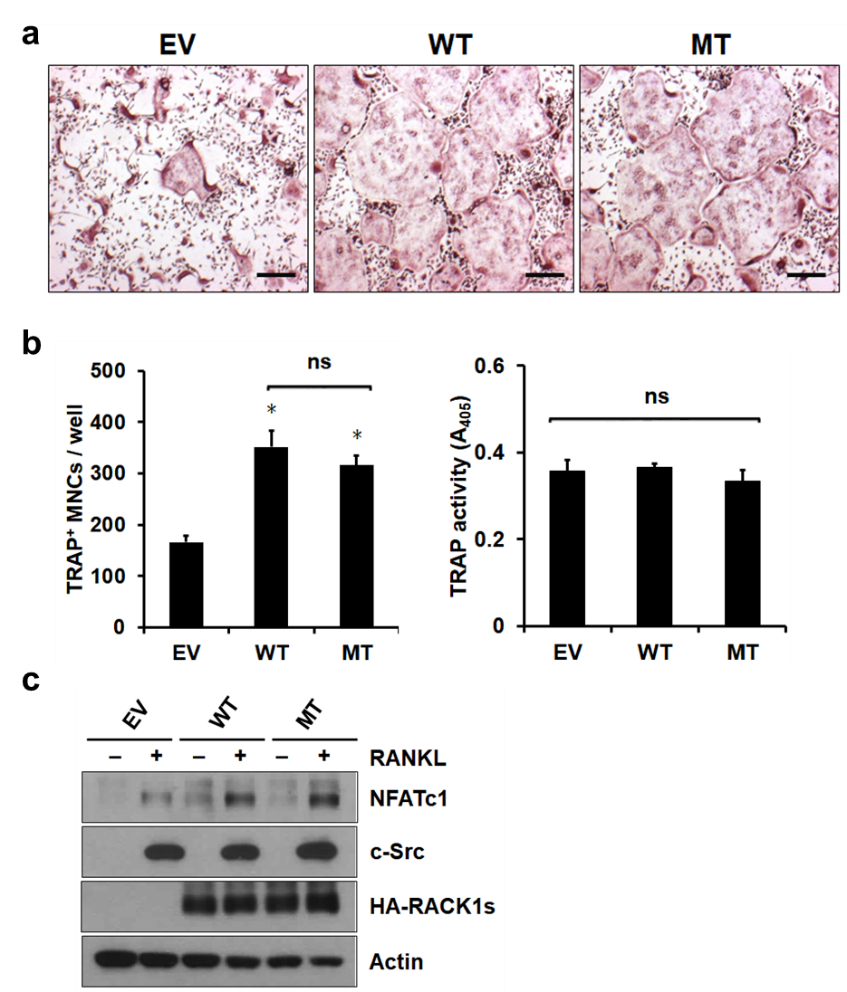


**Supplementary Figure S1.** Both WT-RACK1 and mutant (MT)-RACK1 promote RANKL-induced osteoclastogenesis. BMMs transduced with pMX-puro empty vector (EV), pMX-puro-WT-RACK1 (WT), or pMX-puro-MT-RACK1 (MT) were cultured for 3 d with 30 ng/mL M-CSF in the presence or absence of 100 ng/mL RANKL. (a) Cultured cells were fixed and stained for TRAP. Scale bar, 100 μm. (b) The number of TRAP^+^ MNCs was counted, and TRAP activity was analyzed. A_405_, absorbance at 405 nm. (c) Whole cell lysates were analyzed using Western blot with antibodies against anti-NFATc1, anti-c-Src, anti-HA, and anti-actin. Data are presented as the mean ± SD of three independent experiments. Statistical analyses were done using Student’s *t*-test (**P* < 0.01, ns, not significant).


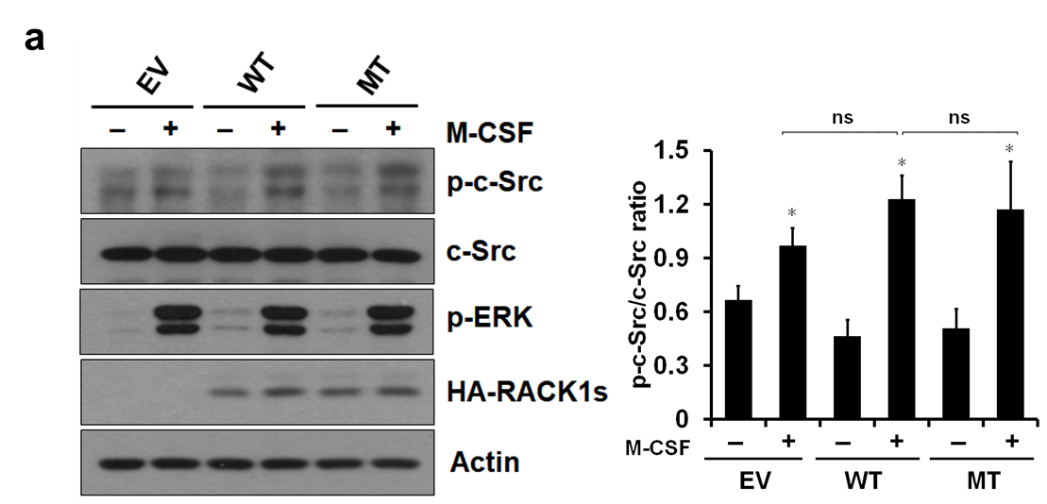


**Supplementary Figure S2. RACK1 does not affect the c-Src activation induced by M-CSF.** (a) Mature osteoclasts transduced as in Fig. 3a were starved and stimulated with 30 ng/ml M-CSF for 20 min. Whole cell lysates were analyzed by Western blotting with antibodies against anti-phospho-c-Src, anti-c-Src, anti-phospho-ERK, anti-HA, and anti-actin. The ratio of p-c-Src to c-Src was quantified from three independent experiments. **P* < 0.01. Western blots are representative of three independent experiments.

**Supplementary methods**

**Reagents**

Recombinant human M-CSF was purchased from R&D Systems (Minneapolis, MN, USA). RANKL was obtained from Peprotech EC (London, England). The antibody against RACK1 used for Western blotting was purchased from BD Biosciences (San Jose, CA, USA). Anti-c-Src was purchased from Abcam Biotechnology (Cambridge, UK). Anti-phospho-c-Src, anti-phospho-ERK and anti-HA were purchased from Cell Signaling Technology (Beverly, MA, USA). The anti-RACK1 antibody used for immunoprecipitation, as well as anti-NFATc1, and anti-β-actin were obtained from Santa Cruz Biotechnology, Inc (Dallas, TX, USA).

**Retrovirus preparation.**

To prepare retroviruses, pMX-puro empty vector, pMX-puro-WT-RACK1 and pMX-puro-MT-RACK1 were respectively transfected into the PLAT-E packaging cell line using using the PEI transfection reagent (Sigma-Aldrich). The retroviruses were used to infect BMMs as previously described. The pMX-puro vector and PLAT-E cells were kindly provided by T. Kitamura (University of Tokyo, Tokyo, Japan). After infection, the BMMs were cultured overnight, detached with trypsin/EDTA, and further cultured in the presence of 30 ng/mL M-CSF and 2 μg/mL puromycin for 2 d. Puromycin-resistant BMMs were induced to differentiate by culturing with 30 ng/mL M-CSF and 100 ng/mL RANKL for an additional 3-4 d.

***In vitro* osteoclast differentiation.**

The cells were fixed and stained for tartrate-resistant acid phosphatase (TRAP) using a TRAP staining kit (Sigma-Aldrich). Osteoclast-like cells were defined as pink TRAP-positive multinucleated cells (i.e., more than three nuclei). The results of the osteoclast formation assays represent the mean of three independent experiments done in triplicate ± standard deviation (SD) of the mean.
